# Exploring six successful nurse-led transition clinics: Experiences and outcomes

**DOI:** 10.1016/j.hctj.2024.100071

**Published:** 2024-08-30

**Authors:** Jobert Sturm, AnneLoes van Staa, Johanna C. Escher, Jane Sattoe

**Affiliations:** a Research Centre Innovations in Care, Rotterdam University of Applied Sciences, Rotterdam, the Netherlands; b Department of Paediatric Gastroenterology, Erasmus University Medical Center, Sophia Children’s Hospital, Rotterdam, the Netherlands

**Keywords:** Transitional care, Young adults, Nurse-led transition clinics, Healthcare transition, Chronic health conditions, Quality standard, Experiences

## Abstract

**Background and purpose:**

In the Netherlands, the 2022 Quality Standard 'Youth in transition from paediatric to adult care' underscores the importance of structured transitional care for young adults with chronic health conditions. Despite this emphasis, detailed knowledge about transition programs and their successful elements remains sparse. This study aims to bridge this gap by exploring nurse-led transition clinics that had successfully implemented core interventions such as a transition coordinator, warm handover, and individual transition plans.

**Methods:**

Employing a mixed-methods approach, this study integrated semi-structured interviews with 15 healthcare professionals from both paediatric and adult care across six transition clinics, and surveys from 54 young adults who had transitioned within the last three years. The ‘On Your Own Feet Framework’ guided the evaluation of transitional care practices. Thematic analysis was applied to qualitative data, while descriptive and inferential statistics were used to analyse quantitative data.

**Results:**

The study revealed a strong dedication among healthcare professionals to ensuring smooth transitions and effective collaboration between paediatric and adult care. The young adults reported high satisfaction with their transitions, particularly appreciating the continuity of care and the pivotal role of nurses and nurse practitioners as transition coordinators. However, challenges such as engaging young adults, resource allocation, and financial complexities were noted, alongside areas for improvement including shared decision-making and managing parental involvement. Motivation and collaboration among staff were identified as facilitating factors.

**Discussion and conclusion:**

Our findings emphasize the vital role of nurse-led transition clinics in enhancing healthcare transitions for young adults in the Netherlands, aligning with the principles outlined in the Quality Standard and the On Your Own Feet Framework. While high satisfaction levels with current practices suggest a positive impact, they also highlight that ongoing improvement and adaptation are needed to overcome identified challenges. Successful healthcare transition requires a comprehensive, collaborative approach involving patients, families, and healthcare professionals, supported by organizational and systemic frameworks. This study contributes to a nuanced understanding of transitional care, suggesting a path forward for integrating these practices into standard care models.

## 1 Introduction

In the Netherlands, over 1.3 million children, adolescents, and young adults (YAs) live with a chronic health condition.^1^ This demographic faces a critical juncture in their healthcare journey: the transition from paediatric to adult care. This healthcare transition is a complex process that includes preparation and transfer, but also post-transfer integration within the adult healthcare system.

This phase is characterized by an abundance of challenges, including having to adapt to a new healthcare environment, gaining independence in health management, and coping with the psychological impact of changes.^2^ Challenges like these can lead to reduced healthcare utilization, medication non-adherence, discontinuation of care, and complications of chronic conditions, resulting in a poorer health status.^3^, ^4^, ^5^, ^6^, ^7^, ^8^

Recognizing the unique needs of YAs during this transition is crucial. The 'On Your Own Feet (OYOF) Framework,' developed by van Staa et al.,^9^ presents a structured approach to transitional care. It emphasizes critical interventions aimed at improving care organization, promoting independence and self-management, and encouraging collaborative efforts between young individuals, their families, and healthcare professionals. In effectively guiding YAs and families through transition to adult care, nursing professionals’ expertise supports crucial patient centred interventions.^10^, ^11^, ^12^

Although relevant frameworks and interventions exist, their practical application leaves much to be desired. Descriptions of these interventions are scarce, and consensus among healthcare professionals regarding the most effective practices is lacking.^13^ Additionally, limited empirical research assessing the impact of such interventions is available.^14^, ^15^, ^16^ This underscores the importance of consolidating existing information on interventions designed to support YAs in their transition from paediatric to adult healthcare. It also highlights the necessity for continued research and further development of policies and practices that can enhance healthcare transition programs for YAs during this critical period. ^17^.

The NICE guideline^16^ and the Dutch 2022 Quality Standard 'Youth in transition from paediatric to adult care'^18^ recommend several interventions aligned with the OYOF Framework. A key element is the role of a transition coordinator, typically a nurse or nurse practitioner, who manages the transitional care.^16^, ^19^ This coordinator is responsible for establishing an individual transition plan that covers medical, care-related, self-management and psychological aspects, guides YAs and their families through the transition, and encourages active participation and discussion.^20^, ^21^, ^22^ Additionally, a critical intervention to ensure continuity of care is the warm handover of care at the actual moment of transfer, involving joint consultations with paediatric and adult healthcare professionals. These warm handovers, along with a first double-time consultation in adult care, are essential in establishing the YA patient’s trust, a fundamental element of treatment adherence.^18^, ^23^

Despite the establishment of guidelines and encouraging reports from local interventions,^24^ disparities still exist in the Dutch healthcare system in the level and quality of transitional care among institutions and medical specialties.^8^ Transition clinics (TCs) represent a proactive response to these disparities, offering multidisciplinary, outpatient care for YAs preparing for the transition to adult healthcare services.^25^ Although TC structures may vary,^26^, ^27^ emerging evidence suggests their potential in facilitating positive transition experiences and enhancing YA satisfaction.^28^, ^29^, ^30^

Nevertheless, detailed knowledge about the specific operations, structure, and valued elements of TCs remains limited. This study aims to fill this gap by investigating nurse-led TCs. It explores the experiences and perceived added value of TCs from the perspectives of nurses, nurse practitioners, and medical doctors across both paediatric and adult care settings, as well as from YAs within these settings. Furthermore, it identifies the practical implementation of key healthcare transition interventions, including the facilitating factors and barriers that impact these implementations. This study seeks to identify the successful elements of TCs that could inform broader practices and policies.

## 2 Methods

### 2.1 Study design

We employed a mixed-methods approach through the concurrent collection and analysis of qualitative and quantitative data. This approach is noted for its ability to triangulate data, thereby enhancing the reliability and validity of findings. By combining the findings from interviews with healthcare professionals and surveys among YAs, we achieved a multifaceted view of the healthcare transition processes, including the identification of facilitating factors and barriers to implementation. The research protocol for this study received approval from the Medical Ethics Review Committee of the Erasmus University Medical Center, with the approval number MEC-2014–246 and an addendum (#2) issued in September 2021.

### 2.2 Selection procedure and sample

Through a preliminary survey in 2021, employing internet searches and chain-referral sampling, we identified 76 TCs in Dutch hospitals. All were sent a questionnaire, with responses received from 51. In the questionnaire, TCs were also asked if they thought their own clinic was a good example of providing effective transitional care. 35 out of 51 TCs responded affirmatively. Of these, nine TCs met the selection criteria, which included being a nurse-led TC and reflecting the presence of recommended transition care interventions. These criteria included the employment of a nurse or nurse practitioner as a coordinator, the development and use of an individualized transition plan for each YA, and the implementation of a warm handover of care involving joint consultations with paediatric and adult healthcare professionals at the actual moment of transfer. To ensure a mix of both established and innovative care practices in different settings, the selection process additionally considered the types of medical conditions treated, the academic or non-academic level of the institutions, and the years of experience with the TC. Eventually, six clinics were purposefully selected because the others were already involved in other research projects.

**Participants**.

Participants were selected from these clinics based on the following criteria:

• **Healthcare professionals**: Medical doctors, nurse practitioners, and nurses involved in TCs, with at least one year of experience in transitional care. The aim was to include a diverse range of experiences and perspectives across both paediatric and adult care specialties. Informed consent was obtained from all participants.
• **YAs**: Healthcare professionals from the TCs selected and approached the YAs based on the following criteria: individuals aged 18-23 years who had transitioned from paediatric to adult healthcare within the past three years, specifically including those diagnosed with the primary conditions treated in the selected clinics. They were contacted via email and, if necessary, received a reminder email. All participants were required to provide informed consent and be capable of engaging with the questionnaire. The YAs were recruited based on a full sample approach.

### 2.3 Data collection

**Interviews with healthcare professionals**.

From October 2021 to January 2022, semi-structured interviews were conducted with fifteen healthcare professionals from both paediatric and adult care settings in the six TCs in five hospitals. These participants were selected for their specific roles in transitional care and their experience working in TCs. Due to the COVID-19 pandemic, these one-hour interviews were conducted online. The interview framework was developed from literature reviews and expert consultations to ensure a comprehensive exploration of transition care topics.

Interview structure and focus areas:

• Organisation and structure: We explored perspectives on effective transitional care, focusing on the operational dynamics of TCs and the coordination between paediatric and adult healthcare services.
• Content and practices: Here, we examined the topics discussed with YAs and their parents during the transition process. This section emphasized healthcare and social participation issues, critically evaluating the rationale of different interventions in transition care.
• Experiences: We aimed to capture the perceived outcomes and benefits of TCs from the viewpoints of healthcare professionals, identifying factors that either impede or facilitate the functioning of TCs.

Ethical considerations were a priority throughout the research process. In compliance with the guidelines set by the Medical Ethics Review Committee and Dutch law, participants were fully informed about the study's objectives, their rights, and the use of their data. Confidentiality was ensured by anonymizing participant data and securely storing all research materials. All interviews were recorded with explicit consent from the participants and transcribed verbatim to ensure accuracy in subsequent data analysis.

**Survey among YAs**.

• **Experiences of YAs with their transition:** Data were collected using the “On Your Own Feet - Transition Experience Scale” (OYOF-TES) (Cronbach's alpha = 0.92),^31^ which measures key aspects of transition, including preparation, readiness, the alliance between paediatric and adult care, reception in adult care, and YA involvement. This validated instrument comprises 20 statements that allow respondents to express their level of agreement on a five-point Likert scale, ranging from 'strongly disagree' to 'strongly agree.' Additionally, overall satisfaction with the transition process is rated on a numerical scale from 1 (very dissatisfied) to 10 (very satisfied), and trust in healthcare professionals is assessed on a scale from 1 (no trust) to 10 (complete trust), separately for paediatric and adult settings.
  To complement the OYOF-TES, our survey explored YAs' broader perceptions and satisfaction with the transition, including:
• **Detailed Satisfaction Assessment:** Satisfaction with paediatric care (7 items, Cronbach's alpha = 0.94), and adult care (6 items, Cronbach's alpha = 0.75) was rated on a five-point Likert scale from "very dissatisfied" to "very satisfied." This scale was developed by our research group in collaboration with experts, based on our extensive experience in transitional care. The perceived role and effectiveness of nurses and nurse practitioners in supporting the healthcare transition process was assessed through a series of 15 newly developed items (Cronbach's alpha = 0.95), also rated on a five-point Likert scale. In a literature review of the role of nursing professionals in transition care, six important roles were identified: advocate, clinician, coach (self-management support), service coordinator, communicator and educator/health promotor.^32^ Based on these roles 15 items were formulated to ask about satisfaction with the role of the nurse or nurse practitioner in transitional care. These 15 items were reviewed on clarity and relevance by young people with chronic conditions (n = 4) and nurse practitioners (n = 4) separately. Based on their feedback the items were finalized.
• **Qualitative Assessments:** Open-ended questions gathered detailed experiences, highlighting the best and worst aspects of their transition.

### 2.4 Data analysis

The data analysis was conducted separately for qualitative and quantitative strands, and then integrated to offer a comprehensive view of the transition process.^33^

**Qualitative data analysis**.

**Fig. 1:**
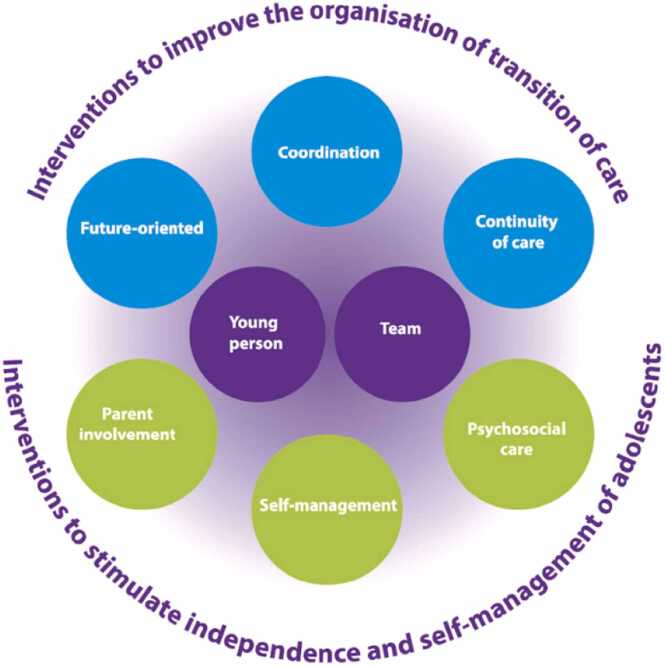
The on your own feet framework.

Qualitative data from semi-structured interviews with healthcare professionals were analysed using ATLAS.ti 23 software. We applied Hsieh and Shannon’s directed content analysis approach^34^, which starts with predefined codes based on the OYOF Framework (see Fig. 1) to direct the analysis, but also allows for the discovery of new themes, ensuring that the analysis remains both focused and flexible. The qualitative data analysis was coordinated and conducted by the first author (JS). All authors were involved in evaluation and interpretation of the data.

• **Initial coding:** We began with a set of pre-defined codes derived from the OYOF framework,^19^ focusing on transition care practices, patient and caregiver experiences, and the roles of various healthcare professionals in the transition process. This deductive coding helped us to systematically organize the data in alignment with our research objectives.
• **Inductive coding:** As the analysis progressed, we remained attentive to emerging themes not previously identified, allowing for inductive coding. This approach ensured that our analysis was responsive to the data, capturing nuances and insights beyond our initial conceptual framework.
• **Thematic analysis:** Through iterative rounds of coding, we identified recurring patterns and themes across the data set. To ensure coherence and consistency, this involved constant comparison, where data segments were compared with each other and with evolving themes.
• **Integration and synthesis:** The final step involved synthesizing the themes into a coherent narrative that encapsulated the complexities of the healthcare transition process. To present a balanced and comprehensive view of the experiences, particular attention was paid to divergent perspectives, conflicts, and consensus among participants.

**Quantitative data analysis**.

Quantitative data analysis was executed using SPSS version 26. We described continuous variables using means and standard deviations, while categorical variables were depicted through frequencies and percentages. The analysis extended beyond descriptive statistics to include inferential statistical techniques. The analyses aimed to understand the relationships between key factors associated with the transition process, as measured by the OYOF-TES and the overall satisfaction and trust levels reported by the YAs.

We commenced our analysis by calculating descriptive statistics to provide an overview of the demographic characteristics of the survey respondents. This included frequencies and percentages for categorical variables such as sex, TC, highest completed education, employment status, and housing situation. For continuous variables such as age, age at the time of transfer, and satisfaction scores, we computed mean values and standard deviations to describe the central tendency and dispersion of these variables.

To explore the relationships between YAs' satisfaction with the transition process and factors such as perceived alignment between care settings, reception in adult healthcare, readiness for transfer, preparation, and shared decision-making, we utilized Pearson's correlation coefficient (r). This analysis aided in determining the strength of association between these factors and satisfaction scores. Correlation coefficients were considered small (*r* = .10), medium (*r* = .30), and large (*r* = .50), based on conventional thresholds.^35^ The significance levels were set at p < .05 to determine the statistical significance of the correlations.

Additionally, we assessed the correlation between trust in healthcare professionals and specific practices within paediatric and adult care settings, including advocacy for the young person’s interests, attentiveness to daily experiences, team collaboration, active involvement in decision-making, and quality of reception in healthcare. Pearson's correlation coefficient (r) was also used for this analysis, with significance levels set at p < .05 to identify statistically significant relationships.

## 3 Results

This section presents an overview of the healthcare settings and participants, followed by a comparative description of the TCs, highlighting their approaches, similarities, and variances. The results are organized into several subsections: an introduction to the departments and interview participants (3.1), a comparative analysis of the TCs for YAs (3.2), the added value of TCs according to healthcare professionals (3.3), facilitating factors and barriers for implementation (3.4), and finally, the results of the young adults’ survey (3.5).

### 3.1 Departments and interview participants

Our research encompassed six TCs located in five hospitals across the Netherlands, including three academic medical centres and two non-academic teaching hospitals. These outpatient clinics address haemophilia, sickle cell disease, inflammatory bowel disease, endocrinological disorders, type 1 diabetes, and immunological disorders. The year of establishment of each TC varied from 2007 to 2016. Each adopted distinct approaches to transitional care for YAs. All invited healthcare professionals participated in the study – both from paediatric and adult care. The demographics and professional backgrounds of the healthcare professionals involved in this study are outlined in Table 1.

**Table 1:** Overview of Health Care Professionals and their expertise.

TC	Academic Center	Disorder	Care Setting	Healthcare Professionals (years of experience)
1	Yes	Haemophilia	Both Both	HCP 1 Nurse Practitioner (14) HCP 2 Nurse (2.5)
2	Yes	Sickle cell disease	Adult Paediatric	HCP 3 Nurse Practitioner (1.5) HCP 4 Nurse (13)
3	No	IBD	Adult Paediatric	HCP 5 Nurse Practitioner (5) HCP 6 Paediatrician (5)
4	Yes	Endocrinological disorders	Paediatric Adult	HCP 7 Nurse Practitioner (3) HCP 8 Nurse Practitioner (5)
5	No	Type 1 diabetes	Paediatric Adult Paediatric Adult	HCP 9 Nurse (10) HCP 10 Nurse (10) HCP 11 Paediatrician (10) HCP 12 Internist (10)
6	Yes	Immunological disorders	Paediatric Paediatric Adult	HCP 13 Nurse Practitioner (1) HCP 14 Paediatrician (6) HCP 15 Nurse Practitioner (6)

TC= transition clinic, HCP= health care professional, IBD= inflammatory bowel disease, Both= HCP healthcare is active in both paediatric and adult care

### 3.2 Comparative analysis of TCs for YAs

We initially identified that all TCs shared essential similarities in their methodologies for managing the healthcare transition of YAs from paediatric to adult care. Notably, in every clinic a nurse or nurse practitioner acted as a coordinator, developed an individualized transition plan, and implemented a warm handover of care at the actual moment of transfer, thereby aligning with our selection criteria. Nevertheless, variations in the execution of these strategies across individual clinics came to the fore. This diversity underscores the range of practices and challenges encountered in the transition phase, highlighting the criticality of tailored approaches within the TC framework.

#### 3.2.1 Similarities across transition clinics

The selection of TCs was based on the implementation of specific nurse-led interventions, crucial for an effective healthcare transition from paediatric to adult care. Given this criterion, the clinics shared several core similarities aimed at a person-centric approach. Individual transition plans, which were detailed and personalized, met the specific needs and conditions of each YA.^24^ Multidisciplinary team consultation meetings and early educational sessions were standard, preparing patients and their families for the transfer to adult care. Nurses and nurse practitioners in their role as transition coordinators focused on education, enhancing treatment adherence, and guiding YAs towards independence.

#### 3.2.2 Differences across transition clinics

Despite these commonalities, transition programs exhibited notable differences that reflected the specific requirements and challenges of each medical condition. The initiation ages for transition processes varied, reflecting adaptability to developmental stages and patient needs. The preparations for transfer, including the timing of independent consultations and educational focuses, were customized. Furthermore, the transfer process itself differed from clinic to clinic, with some facilitating joint consultations and others organizing warm handover sessions. The reception in adult care also varied, featuring different follow-up consultation frequencies and digital tool usage for ongoing care and communication, underscoring the commitment to tailored care for unique patient challenges during the transition.

Interventions to enhance participation and collaboration, interventions to improve the organization of care, and interventions to stimulate independence and self-management occur at all levels and stages and vary among TCs. The specific characteristics and methodologies of TCs, summarized in Table 2, detail these key characteristics and methodologies.

**Table 2:** characteristics of Transition Clinics.

	Haemophilia	Sickle cell disease	IBD	Endocrinological disorders	Type 1 Diabetes	Immunological disorders
Establishment year TC	2007	2009	2016	2017	2011	2015
**Age of starting transition process**	12	12	12	14	12	11
**Preparations for transfer*****(to stimulate independence and self-management)***	MDT consultations at ages 4, 12, and 17Educational sessions between ages 4 −15Two consultations per year with nurse and paediatrician/nurse practitioner between ages 12 −20ITP and Quality of life assessment starting at age 12Independent consultations starting at age 15	MDT consultations at ages 4, 12, and 16Educational sessions between ages 8 −16ITP starting at age 17.5Independent consultations starting at age 16	Transition brochureITP starting at age 12YA as primary contact starting at age 16 −17Quarterly and independent consultations with paediatric gastroenterologist and adult nurse practitioner at paediatric outpatient clinic starting at age 17Intravenous medication administration in adult care starting at age 17.5	Biannual consultations with nurse practitioner and paediatrician (including education, self-management and adherence) ITP starting at age 14 Independent consultations before transfer	Quarterly half-hour consultations, educational meetings ITP and Quality of life assessment starting at age 12 Preparation folder at age 16 Independent consultations before transfer	Annual educational consultations starting at age 12 ITP starting at age 12 Independent consultations starting at age 16
**Transfer*****(to enhance participation and collaboration)***	Warm handover: last consultation with paediatrician, first consultation with haematologist	Warm handover: two consultations 1) getting acquainted with the adult doctor at paediatric outpatient clinic 2) saying goodbye to the paediatrician at adult outpatient clinic Nurse walks with YA from paediatric clinic to adult clinic during farewell	Warm handover: one consultation between paediatric gastroenterologist and gastroenterologist Already familiar with adult nurse practitioner	Warm handover between nurse practitioners paediatric and adult care One-time consultation with all involved HCPs	Warm handover: two consultations preceded by MDT with all involved HCPs First consultation at paediatric outpatient clinic Second consultation: three months later at adult outpatient clinic	Warm handover: two MDT meetings at adult outpatient clinic between involved HCPs followed by consultation with paediatric and adult care HCPs
**Reception in adult care*****(to stimulate independence and self-management and to enhance participation and collaboration)***	Consultations focusing on treatment and guidance for YAs	Consultations every six months Follow-up call after second visit to adult care	Alternating consultations with nurse practitioner and gastroenterologist Ongoing support	Early follow-up with nurse practitioner 4 −6 months post-transferConsultations with endocrinologist	Half-yearly consultations Use of digital tool (freestyle libre)	Consultations with an initial follow-up after three months
**HCPs involved*****(to improve the organization of care)***	Paediatric Care: Paediatric haematologist, nurse, nurse practitioner, physiotherapist, social worker, psychologist Adult Care: haematologist, nurse, nurse practitioner, physiotherapist, social worker, psychologist	Paediatric Care: Paediatric haematologist, nurse, nurse practitioner, psychologist, social worker Adult Care: haematologist, nurse, nurse practitioner	Paediatric care: nurse, paediatric gastroenterologist Adult care: nurse, gastroenterologist, nurse practitioner	Paediatric Care: Paediatric endocrinologist, nurse practitioner, psychologist Adult Care: Endocrinologist, nurse practitioner, psychologist	Paediatric Care: Paediatrician, nurse, dietitian Adult Care: Internist, nurse, dietitian	Paediatric Care: Paediatric immunologist, nurse practitioner Adult Care: Immunologist, nurse practitioner
**Role nurse/nurse practitioner*****(to improve the organization of care and to stimulate independence and self-management)***	Transition coordinator Education, adherence and treatment	Transition coordinator Education, adherence and guidance to independence	Transition coordinator Education and supporting self-management	Transition coordinator Guidance and disease control	Transition coordinator Guidance and disease control	Transition coordinator Guidance and treatment
**Parental involvement*****(to stimulate independence and self-management)***	Guidance from birth (educational sessions) Reduced over time, attend appointments until age 15 Guidance in passing on responsibility	Reduced over time, attend appointments until age 16 Guidance in gradually letting go	Encouraging YA take responsibility Enhancing parental trust	Active role until age 18, gradual reduction Guidance in reducing caregiving responsibilities Questionnaires for parents	Active role until age 18, gradual reduction Guidance in reducing caregiving responsibilities Questionnaires for parents	Gradual reduction, focus on YA independence Guiding parents to let YA take charge
**Coordination, continuity & communication*****(to improve the organization of care)***	MDT meetings every six weeks Shared physical workspaces Involvement of HCPs known from paediatric settings in adult care Clinical pathway (transition protocol)	Follow-ups Close collaboration between paediatric and adult care HCPs Clinical pathway (transition protocol)	Weekly MDT consultations Communication among HCPs Clinical pathway (transition protocol)	Close collaboration and joint consultations between paediatric and adult care HCPs Clinical pathway (transition protocol)	Close collaboration and joint consultations between paediatric and adult care HCPs Clinical pathway (transition protocol)	Close collaboration and joint consultations between paediatric and adult care HCPs Clinical pathway (transition protocol)

IBD= inflammatory bowel disease, YA= young adult, ITP= individual transition plan, MDT= multi-disciplinary team, TC= transition clinic, HCPs= health care professionals

### 3.3 The added value of TCs according to healthcare professionals

Three core themes emerged from our analysis: patient empowerment, family support, and care coordination. These themes demonstrate how TCs contribute to a more effective transition process for YAs from paediatric to adult healthcare.

#### 3.3.1 Patient empowerment

Healthcare professionals consistently highlighted the role of TCs in enhancing patient empowerment. Training in self-management skills was a central component across all clinics, enabling YAs to take greater control over their health. For instance, structured educational programs and the use of tools, such as the "Ready Steady Go" programme, appeared to increase patients' confidence in managing their conditions more independently. YAs reported greater self-reliance and an improved ability to navigate their healthcare needs post-transition. According to professionals, this emphasis on empowerment seems to contribute to better health outcomes and fosters a sense of autonomy among YAs, which is crucial for their long-term well-being.

#### 3.3.2 Family support

The transition process also involved notable support for families, which healthcare professionals deemed important. TCs aimed to provide tailored guidance to parents, helping them shift their roles from primary caregivers to supporters. This gradual reduction in parental involvement was managed through educational sessions and consistent communication, aiming to prepare families for the transition. For example, in the haemophilia clinic, efforts were made to engage parents in the transition process from the onset, which helped ease the handover of responsibility to YAs. Emotional support strategies for families were also employed to facilitate smoother transitions and potentially improve health outcomes for YAs. Nonetheless, healthcare professionals have highlighted that there is still room for improvement in supporting parents throughout this process.

#### 3.3.3 Care coordination

Effective care coordination emerged as a critical theme, with seamless communication among healthcare teams being a standout feature. Collaborative efforts between paediatric and adult care providers ensured continuity of care, enhancing patient satisfaction according to professionals. For example, in the sickle cell disease and endocrinological disorder clinics, MDT meetings and shared clinical pathways were employed to align treatment plans and protocols. This unified approach not only prevented gaps in care but also facilitated a smoother transition experience for YAs. Continuous collaboration and the use of integrated electronic health records were also highlighted as significant factors in maintaining coordinated care.

Table 3 presents an overview of the perceived added value of TCs across various health conditions as reported by healthcare professionals.

**Table 3:** Added value of transition clinics according to healthcare professionals.

	Haemophilia	Sickle cell disease	IBD	Endocrinological disorders	Type 1 Diabetes	Immunological disorders
**Patient empowerment**	Training in self-management skills leads to measurable increases in patient self-reliance and ability to manage condition independently.	Initiatives promoting self-management directly link to enhanced patient empowerment, with patients reporting greater confidence in managing their health post-transfer.	Early engagement strategies significantly increase YAs’ readiness and awareness for transition, leading to improved self-management outcomes post-transition.	Increasing YAs' awareness and readiness for managing their condition through targeted educational programs, results in improved independence and self-management.	Focused educational programs and the use of digital tools effectively encourage patient independence, with patients showing improved management of their condition.	The program supports autonomy through structured educational sessions, leading to significant improvements in patient self-management and empowerment
**Family support**	From birth, parents receive tailored guidance, enhancing their capacity to transition support roles effectively, evidenced by increased patient independence over time.	Emphasized parental understanding of disease complexities results in better-prepared families, contributing to smoother transitions as reported by families.	Emotional support strategies for families have been shown to reduce transition-related stress, contributing to more positive health outcomes for YAs.	Preparation of parents to adapt to and support their child’s transition has led to improved autonomy and self-care among YAs.	Active involvement of parents until age 18 in caregiving responsibilities results in a smoother transition, with YAs better prepared to take on self-care responsibilities.	Focused on gradual reduction of parental involvement, guiding parents to let YAs take charge has led to successful transitions marked by patient self-efficacy.
**Care coordination**	Demonstrated seamless communication among teams results in fewer transitional errors and improved patient satisfaction.	A unified approach ensures that no patient falls through the cracks during the transition, as evidenced by continuity in care and treatment adherence.	Tailoring of care protocols to individual needs produces flexibility, resulting in higher patient and family satisfaction with the transition process.	Ensured smooth transition and continuity of care leads to reduced gaps in care during the transition phase.	Close collaboration and use of digital tools for continuity and communication significantly improve care coordination.	Continuous collaboration between care teams ensures that transitions are smooth, with patients reporting a seamless experience.

IBD= inflammatory bowel disease, YAs= Young adults

These findings underscore the multifaceted value of TCs in facilitating transitions from paediatric to adult healthcare.

### 3.4 Facilitating factors and barriers for implementation

The successful implementation of transitional care programs is influenced by different facilitators and barriers. This section explores these elements to provide a comprehensive view of what supports or hinders the smooth transition from paediatric to adult care.

#### 3.4.1 Facilitating factors

Several key factors facilitate the effective implementation of transitional care programs. Foremost among these is the strong motivation of staff, complemented by the active involvement of both paediatric and adult care teams, which underscores the collaborative spirit as being essential for transitional care success. The tailored application of protocols across departments, integration of transitional care programs into electronic patient records, and the backing of transitional care by management through supportive organizational frameworks further facilitate this process. Professional development, especially training nurses in transitional care, and the engagement of patients and parents through feedback mechanisms, are facilitators, too. Table 4 provides specific examples from healthcare professionals illustrating these facilitating factors.

#### 3.4.2 Barriers

Conversely, several barriers to effective implementation were identified, such as challenges in engaging YAs, attributed to the complexities of adolescence and transitioning identities. Furthermore, time and resource constraints significantly hinder cooperation with adult care facilities, while integration within information systems, crucial for care continuity, remains a challenge. Financial complexities were highlighted as significant impediments, including billing issues for joint consultations and sustaining efforts that are not directly compensated. Additionally, personnel and organizational challenges were noted, such as inefficiencies and inadequate motivation within resource-focused healthcare settings. Table 4 contrasts these challenges with the facilitating factors.

**Table 4:** Facilitating factors and barriers for implementation.

Facilitators	HCP Citations
Motivation and collaboration among staff	HCP5: "Motivation is the key driver for us." HCP7: "It [a transition clinic] can only be established with motivation from professionals on both sides, which is the first step. The second step is collaboration and organizing care well.”
Tailored application of protocols	HCP13: " Each department implements it in its own unique way, yet with consistent dedication."
Integrated electronic patient record	HCP10: “Initially, we couldn't view the paediatric files, but we requested authorization for that. We arranged to view each other's files.”
Management and organizational structures	HCP4: "Transitional care has been a focal point of our hospital. It's on everyone's agenda, even with changes in management."
Professional development	HCP4: "Training nurses in transitional care has played a crucial role."
Patient and parent involvement	HCP14: "We interviewed YAs pre- and post-transfer for feedback, identifying gaps in support and communication for those over 18, leading to significant improvements in our approach." HCP11: “Most parents appreciate a gradual approach."
Adherence to evidence-based guidelines	HCP8: "Evidence from literature has been a guiding force."
**Barriers**	**HCP Citations**
Challenges in engaging YAs	HCP13: "Some YAs exhibit strong resistance, often influenced by the challenges of adolescence."
Time and resource allocation	HCP11: "Securing time from adult care was a challenge, but it was eventually successful, though it required extra effort."
Integration of information systems	HCP13: "Individual transition plans should be highlighted in the electronic health record for visibility to new healthcare providers."
Financial complexities	HCP3: "I'm unsure about the financials for a joint consultation with two doctors. I believe the adult doctor doesn't bill it, but I'm not certain." HCP15: "For a warm handover, the paediatrician sits pro bono with the internist, because you can’t bill twice. For large patient groups, paediatricians can't work pro bono every month."
Personnel and organizational issues	HCP14: "It's not efficient care, requiring motivation. For financially focused organizations, this inefficiency can be a significant drawback."

HCP= health care professional, YAs= young adults

### 3.5 Results of the young adults survey

A total of 204 individuals were invited to participate in the survey, 55 of whom provided valid responses, resulting in a response rate of approximately 27 %. Non-valid responses were excluded from the final analysis due to issues such as incomplete surveys or ineligibility based on predefined criteria.

#### 3.5.1 Demographics and background characteristics

The demographics of these respondents are depicted in Table 5.

**Table 5:** Background characteristics young adults (n = 55).

	N (%)	Mean ( ± SD)
**Sex (n = 55)**		
Male Female	31 (56.4) 24 (43.6)	
**Age (n = 51)**		
18 19 20 21 22 23	9 (17.7) 11 (21.6) 12 (23.5) 11 (21.6) 4 (7.8) 4 (7.8)	20.04 ( ± 1.48)
**Age at time of transfer (n = 55)**		
17 18 19 20 21 22	9 (16.4) 30 (54.5) 7 (12.7) 4 (7.3) 3 (5.5) 2 (3.6)	18.42 ( ± 1.23)
**Time since transfer in years (n = 51)**		
0 −11 −22 −3	25 (49.0) 11 (21.6) 15 (29.4))	1.69 ( ± 1.09)
**Transition Clinic (n = 55)** Haemophilia/Sickle Cell IBD Endocrinological disorders Type 1 Diabetes Immunological disorders	14 (25.5) 9 (16.4) 15 (27.2) 12 (21.8) 5 (9.1)	
**Highest completed education at time of research (n = 55)**		
Primary school Secondary school Vocational education Bachelor’s degree Master’s degree	5 (9.1) 29 (52.8) 12 (21.8) 7 (12.7) 2 (3.6)	
**Current employment (n = 55)**		
Yes, paid Yes, unpaid (volunteer work) No	39 (70.9) 3 (5.5) 13 (23.6)	
**Current housing situation (n = 55)**		
Independent With parents Other	8 (14.5) 46 (83.7) 1 (1.8)	

#### 3.5.2 Satisfaction and trust scores

**Fig. 2:**
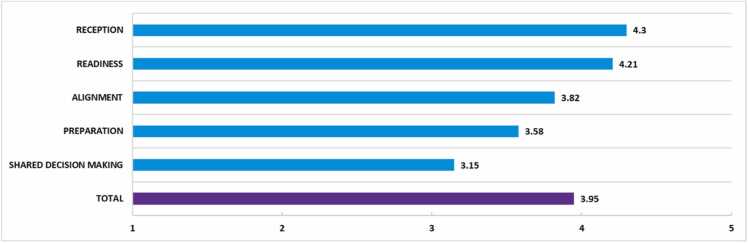
Average total and subscale scores on the OYOF-TES (range 1–5).

The mean satisfaction score of 7.8 ( ± 1.5) on a Visual Analogue Scale ranging from 1 to 10 reflects a generally positive experience among YAs with the overall transition process. However, the mean sub-scales of the OYOF-TES related to the alignment between paediatric and adult care and the preparation for transfer received comparatively lower satisfaction scores. Due to the small sample sizes, it was not possible to test for differences in transition experiences across various transition clinics. YAs reported less positive experiences particularly in their involvement in decision-making regarding the timing and location of their transition, suggesting a need for more inclusive approaches (Fig. 2). A higher satisfaction score was strongly associated with well-perceived alignment between paediatric and adult care (r = .75) and a good reception in adult care (r = .68) (p < .01).

YAs’ confidence in healthcare professionals was notably high, demonstrating strong trust across both environments. Employing a scale that ranged from 0 (indicating no trust) to 10 (signifying complete trust), we found that paediatricians were highly trusted, with an average trust score of 8.96 (SD = 1.4). Trust remained significantly high for doctors in adult care as well, as evidenced by a mean score of 8.57 (SD = 1.3).

Similarly, nurse practitioners and nurses in paediatric settings were accorded high trust, with a mean score of 8.75 (SD = 1.3). Although trust scores for nursing professionals in adult care showed a slight dip, they remained robust with a mean score of 8.42 (SD = 1.8).

#### 3.5.3 Detailed satisfaction assessment

**Fig. 3:**
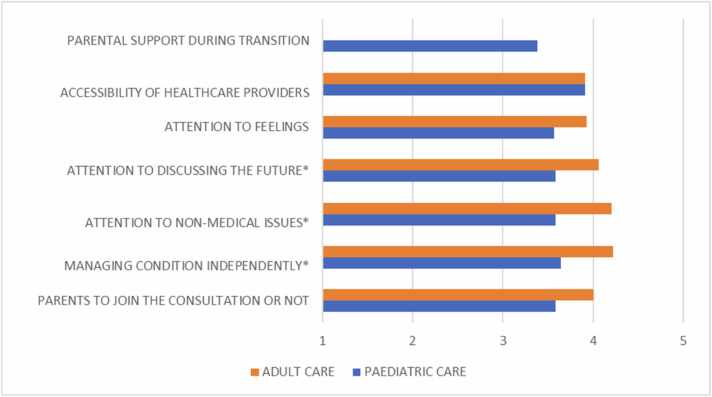
Satisfaction with paediatric and adult care (range 1–5). *statistically significant.

Fig. 3 indicates the areas of satisfaction among YAs in both paediatric and adult healthcare settings. In general, the scores suggest that satisfaction is higher in adult care for most categories compared to paediatric care, except for the accessibility of healthcare providers, which is consistent across both settings. Specifically, adult care scored significantly higher in terms of attention to discussing the future (p = .025), attention to non-medical issues (p = . 001) and learning to manage the condition independently (p = .004).

#### 3.5.4 Feedback on the role of nurses and nurse practitioners

**Fig. 4:**
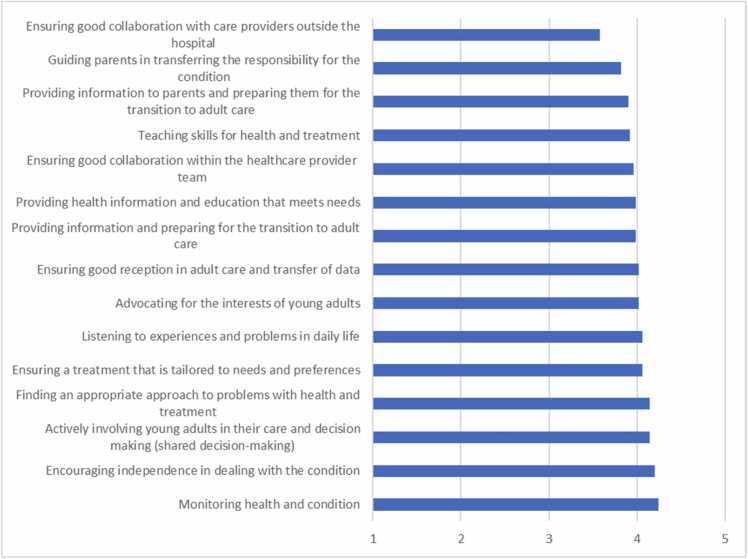
Mean satisfaction of young adults with the role of the nurse/nurse practitioner (range 1–5).

In addition, YAs provided significant positive feedback about the role of nurses and nurse practitioners in facilitating their care transition. The positive feedback related to attention in monitoring health and medical conditions and to encouraging independence in managing these conditions. However, nurses and nurse practitioners were perceived as less effective in facilitating seamless collaboration with external healthcare providers, such as general practitioners, schools, youth teams, and mental health services, and in guiding parents, as depicted in Fig. 4.

Statistical analysis revealed that higher scores of YAs on 'advocating for interests' (r = .54), 'listening to experiences and problems in daily life' (r = .52), and 'ensuring good collaboration within the healthcare provider team' (r = .52) are associated with more trust in the paediatric care nursing professionals (p < .01).

Higher scores of YAs on 'actively involving young adults in their care and decision-making (shared decision-making)' (r = .55), 'advocating for the interests of young adults' (r = .55), and 'ensuring good reception in adult care and transfer of data' (r = .57) were associated with more trust in the adult care nursing professionals (p < .01).

#### 3.5.5 Open-ended survey responses

In this section, we delve into the thematic analysis presented in Table 6, which distils the experiences of YAs transitioning from paediatric to adult healthcare services into the most positive and negative experiences, as reported by the participants themselves. This analysis not only provides personal insights into the transition journey but also underscores the critical factors contributing to a successful or challenging transition.

**Table 6:** Most positive and most negative experiences according to young adults.

Theme	Most positive experiences
Information and preparation	YA IBD: “I was well informed and knew what to expect. I also found both doctors very pleasant.” YA endocrinology: “I notice little difference, the interaction with the doctor is comparable.”
Continuity of care	YA haemophilia: “It was nice that there were familiar faces during the transition, which made the step less daunting for me.”
Smooth transition	YA IBD: “The paediatrician guided me well and walked with me to my new doctor the first time. The whole process was very smooth, and I had nothing to complain about.” YA endocrinology: “It went very smoothly. I felt heard.”
Feeling welcome and at home	YA sickle cell: “I felt welcome and quickly felt at home in the new department because of the doctors and other caregivers.”
Engagement and support	YA type 1 diabetes: “Especially the guidance throughout the entire process was very nice.” YA type 1 diabetes: “The involvement of both departments through joint appointments.” YA immunology: “Good information and a nice team.”
Satisfaction with healthcare professionals	YA immunology: “Everything was super well arranged, and I am very satisfied with my new healthcare provider.”
**Theme**	**Most negative experiences**
Lack of continuity and abrupt transition	YA haemophilia: “My transition to the adult department was very abrupt. I was admitted to the paediatric ward and suddenly had to move to the adult department. I didn't want to make a fuss by asking if I could stay in paediatrics a bit longer because I hadn't been officially transferred yet.”
Communication and information deficit	YA IBD: “It was not communicated that my parents could no longer accompany me during treatment. And that I now had to submit stool samples and have blood drawn before the treatment myself.” YA type 1 diabetes: “When I had a phone consultation scheduled with the doctor, I wasn't called. And I wasn't called later either. I haven't heard anything about it since.”
Loss of personal attention	YA IBD: “During my treatment in adult care, not just one nurse helped me, but multiple, which made the care much less personalized.”
Challenges in navigating adult care	YA endocrinology: “When I turned 18, I had to arrange my own health insurance and therefore my own medication. I knew little about this and consequently spent unnecessary amounts of money on medication.” YA IBD: “Due to scheduling problems, I never really had a handover meeting with my paediatrician and the internist. I found this disappointing at the time.”
Impact of COVID−19 on the transition of care	YA type 1 diabetes: “My transition happened just before the COVID-pandemic, so I have never seen the internist in person. This made contacts with healthcare providers difficult and has made me feel that the transition was not smooth.” YA immunology: “My transition was during the pandemic, which made my transition different from that of my older sister.”

YA= Young adult, IBD= inflammatory bowel disease

Positive experiences such as effective information and preparation, continuity of care, and a welcoming atmosphere in new departments highlight crucial elements of transitional care. These elements should serve as benchmarks for designing and implementing transitional care programs across healthcare settings. Conversely, negative experiences such as abrupt transitions, communication gaps, and a loss of personal attention pinpoint critical areas for improvement.

## 4 Discussion

This study aimed to address knowledge gaps regarding the operations, structure, and successful elements of nurse-led TCs, in managing healthcare transitions for YAs with chronic conditions. Our findings highlights how these can be integrated and optimized within current healthcare practices. Using a mixed-methods approach, we explored participants’ experiences and the perceived added value of TCs, identifying common themes such as patient empowerment, family support, and care coordination. While there were many similarities among clinics, notable variations in the implementation of transitional care emphasized the need for flexible and adaptable strategies. These findings are consistent with the principles of person-centred care advocated by both the NICE guideline^16^ and the Dutch Quality Standard^18^.

Our evidence supports the significance of individual transition plans and warm handovers,^18^, ^20^, ^21^, ^22^ and underscores the effectiveness of nurses and nurse practitioners as indispensable transition coordinators.^11^, ^29^, ^34^ They concentrate on education, enhancing treatment adherence, and guiding YAs toward independence while monitoring their health and conditions. These professionals are tasked with coordinating and organizing transition activities, with a focus on maintaining person-centric care throughout the transitional process.

By comparing the OYOF-TES scores from our study with those from other studies^29^, ^36^, ^37^ and analysing YAs' satisfaction scores, our findings reaffirm the value of transitional care when approached as a continuous, multi-year engagement that begins early and fosters independence. Additionally, ample attention was given to the 'transition of parents' within the TCs studied, emphasizing the importance of supporting parents during their role change.^38^ High satisfaction scores from YAs highlight the perception of healthcare transition as an essential component of standard care.

### 4.1 Limitations

We addressed potential biases inherent in self-reported data through data triangulation. Still, the reliance on self-reports and a relatively low response rate (27 %) among YAs may limit the generalizability of our conclusions. Future research should aim for a broader participant base and incorporate objective measures. The deliberate selection of TCs known for successful implementation implies a selection bias, possibly overlooking other good examples. However, we do not expect that including more examples would have led to different insights.

### 4.2 Implications

Our research highlights successful practices and suggests the need for broader exploration of TC experiences. The findings enhance our understanding of healthcare transition for YAs by reiterating the crucial role of personalized care planning and coordination provided by nursing professionals.^10^, ^11^ The diverse approaches observed in transitional care, indicate that TC models are multifaceted and adaptable to different healthcare systems. This underscores the importance of standardized yet adaptable approaches to healthcare transition, with an emphasis on person-centeredness.

The commitment of dedicated staff and the necessity of interdisciplinary collaboration emerge as pivotal factors in the success of healthcare transitions. These elements are consistent with the core principles of the OYOF Framework and previous research.^29^ However, to streamline healthcare transition delivery, aspects such as effectively engaging YAs, integrating care protocols into existing health information systems, and navigating the financial complexities of healthcare systems^29^ will need to be improved.

High satisfaction scores among YAs reflect the critical role of nurses and nurse practitioners in facilitating smooth transitions. YAs reported high levels of trust and satisfaction with the guidance and monitoring provided by these professionals. Still, areas for improvement were identified, such as enhancing collaboration with community services, including general practitioners, schools, youth teams, and mental health services, and guiding parents during the transition. While the role of nurses and nurse practitioners is vital and well-regarded, there is a need for enhanced coordination and support mechanisms to address these gaps and further improve the transition experience for YAs. Looking forward, we advocate for continued investigation into the long-term impacts of transitional care programs on YAs' health and well-being, as well as the scalability of these models across different healthcare systems. Such research efforts could deepen our understanding of successful transitions and inform comprehensive, evidence-based transitional care guidelines.

## 5 Conclusion

In conclusion, our study underscores the indispensable role of nurse-led TCs in facilitating healthcare transitions for YAs with chronic conditions. By identifying critical success factors, we provide valuable insights for enhancing transitional care and advocate for its integration as a fundamental element of standard care practices.

Our findings substantiate the components of the OYOF Framework with empirical evidence and propose necessary preconditions to realize these elements. For instance, the integration of care protocols into electronic health records is essential for maintaining continuity of care, and navigating billing issues for joint consultations remains a critical challenge.

Our study provides insights into TCs providing high-quality transitional care and the added value they bring. The working ways, transition interventions, barriers, and facilitators give guidance on how to improve transitional care. We suggest adopting these best practices and principles to ensure that transitional care is not only effective but also seamlessly integrated into the continuum of care for YAs with chronic conditions.

## Funding

Funded by the Dutch Organisation for knowledge and innovation in health, healthcare and well-being (ZonMw), grant number 10040012010003 and from a doctoral grant from Rotterdam University of Applied Sciences.

## Ethical statement

The Medical Ethics Research Committee of the Erasmus University Medical Center approved the research protocol (MEC-2014–246; addendum #2, September 2021).

## Ethical statement

1) This material is the authors' own original work, which has not been previously published elsewhere.
2) The paper is not currently being considered for publication elsewhere.
3) The paper reflects the authors' own research and analysis in a truthful and complete manner.
4) The paper properly credits the meaningful contributions of co-authors and co-researchers.
5) The results are appropriately placed in the context of prior and existing research.
6) All sources used are properly disclosed (correct citation). Literally copying of text must be indicated as such by using quotation marks and giving proper reference.
7) All authors have been personally and actively involved in substantial work leading to the paper, and will take public responsibility for its content.

The violation of the Ethical Statement rules may result in severe consequences.

## CRediT authorship contribution statement

**Johanna C. Escher:** Writing – review & editing, Supervision. **AnneLoes van Staa:** Writing – review & editing, Writing – original draft, Supervision, Methodology, Investigation, Conceptualization. **Jobert Sturm:** Writing – review & editing, Writing – original draft, Formal analysis. **Jane Sattoe:** Writing – review & editing, Writing – original draft, Supervision, Project administration, Methodology, Investigation, Funding acquisition, Data curation, Conceptualization.

## Declaration of Competing Interest

The authors declare that they have no known competing financial interests or personal relationships that could have appeared to influence the work reported in this paper.

## Data Availability

Data will be made available on request.
